# Outcomes of thoracic endovascular aortic repair with fenestrated surgeon-modified stent-graft for type B aortic dissections involving the aortic arch

**DOI:** 10.3389/fcvm.2022.1031068

**Published:** 2023-01-16

**Authors:** Xiaoye Li, Lei Zhang, Chao Song, Hao Zhang, Shibo Xia, Yang Yang, Longtu Zhu, Wenying Guo, Qingsheng Lu

**Affiliations:** ^1^Division of Vascular Surgery, Department of General Surgery, Changhai Hospital, Naval Medical University, Shanghai, China; ^2^Changhai Hospital, Naval Medical University, Shanghai, China

**Keywords:** thoracic endovascular aortic repair, fenestrated surgeon-modified stent-graft, type B aortic dissection, aortic arch, supra-aortic trunks

## Abstract

**Objectives:**

This retrospective analysis aimed to evaluate the early and midterm outcomes of thoracic endovascular aortic repair (TEVAR) with fenestrated surgeon-modified stent-graft (f-SMSG) for type B aortic dissections (TBAD) involving the aortic arch.

**Methods:**

From March 2016 to April 2021, 47 consecutive patients were treated using TEVAR with f-SMSG. All patients were diagnosed with TBAD involving the aortic arch.

**Results:**

In total, 47 patients with TBAD involving the aortic arch were treated with f-SMSGs. There were 21 zone 1 and 26 zone 2 TEVAR, and 65 arteries were revascularized successfully with fenestrations. Technical success was achieved in 46 patients (97.88%). The 30-day estimated survival (± SE) and reintervention was 93.6 ± 1.0% (95% Confidence Interval [CI], 92.6–94.6%) and 91.5 ± 1.2% (95% CI, 90.3–92.7%), respectively. During a median follow-up of 51 months (range, 16–71 months), 1 patient died of rupture of aortic dissection (AD) and 3 patients died of non-aortic-related reasons. Reintervention was performed for four patients, including two patients of type IA entry flow and two patients of type IB entry flow. No occlusion of the supra-aortic trunk was observed. The estimated survival and reintervention (± SE) at 4 years was 88.7 ± 1.4% (95% CI, 87.3–90.1%) and 84.8 ± 1.5% (95% CI, 83.3–86.3%), respectively.

**Conclusion:**

Thoracic endovascular aortic repair with f-SMSG is an alternative treatment option for TBAD involving the aortic arch in high-volume centers.

## Introduction

The incidence of aortic dissection (AD) was 4.8 per 100,000 individuals/year, two-thirds of whom presented with type A AD (TAAD) and the remaining one-third with type B (1). Optimal medical therapy (OMT) is recommended for uncomplicated type B aortic dissections (TBAD) without high-risk features. For complicated TBAD and uncomplicated TBAD with high-risk features, thoracic endovascular aortic repair (TEVAR) is recommended as the first-line treatment (2). Considering superior aortic remodeling after TEVAR over OMT in the long-term, TEVAR had been also used in uncomplicated cases (3, 4).

With TEVAR, entry tears of TBAD were completely excluded and blood flow was stopped from entering the false lumen. Adequate length of proximal/distal landing zone is necessary for complete sealing, otherwise, blood flow would re-enter the false lumen, resulting in progression or eventually rupture. For adequate length in the healthy aorta, a proximal landing zone had to be extended proximal to the ostium of the left subclavian artery (LSA), left common carotid artery (LCCA), and even innominate artery (IA). In these conditions, supra-aortic trunks must be revascularized, otherwise, severe complications would occur.

Available options for supra-aortic trunk revascularization included TEVAR with branched and/or fenestrated stentgraft, scallop technique, and bypass surgery (5). In our center, TEVAR with fenestrated surgeon-modified stent-graft (f-SMSG) was mostly used and has been performed for more than 5 years. With this retrospective study, we reported its outcomes for the treatment of TBAD.

## Materials and methods

### Population

Between March 2016 and April 2021, 47 consecutive patients with TBAD involving the aortic arch underwent zone 1/2 TEVAR in our center. Protocol and informed consent were approved by the institutional review board, and all patients gave written consent. Indications for TEVAR with f-SMSGs were patients with TBAD involving the aortic arch (primary entry tear in zone 1 or distal, the proximal extent of pathology in zone 1 or distal). Exclusion criteria were (1) the proximal extent of the pathology/entry tear in zone 0 and (2) whether the maximal aortic diameter of the proximal landing zone was more than 45 mm. Baseline characteristics, images, and operative and follow-up data were prospectively collected and retrospectively reviewed.

### Pre-operative planning and design

Pre-operative computed tomography angiography (CTA) in Dicom format (axial slice thickness of 3 mm or less) of all patients was acquired. The anatomical features were measured with vascular imaging workstation Aquarius (TeraRecon, Foster City San Mateo, CA, USA) or Endosize (Therenva, Rennes, France). All measurements were taken in multiplanar reconstruction always in a plane perpendicular to the manually corrected local aortic centerline. The diameter of the aorta at the proximal and distal landing zone, the diameter and clock position of the Ostia of LCCA and LSA, and the diameter of LCCA and LSA were measured along the centerline. The distance between the Ostia of LCCA and LSA was measured along the greater curvature line. All f-SMSGs were 0–5% oversized to the aorta. In cases where the distal landing zone was the dissected aorta, the diameter of the long axis of the true lumen was used to determine the oversize ratio. For zone 1 TEVAR, two strategies were used, including one large fenestration for LCCA and LSA and two small fenestrations for LCCA and LSA, respectively. For a patient with the right common carotid artery (RCCA), which originated directly from the aortic arch, a large fenestration for RCCA and LCCA was used. For zone 2 TEVAR, the revascularization strategy was a small fenestration for LSA. A large fenestration is defined as a fenestration aligned with more than one artery, while a small fenestration is defined as a fenestration aligned with one artery. When entry tears were on the lesser curvature side, a large fenestration would be selected. Bypass surgery would be performed when supra-aortic trunks were dissected.

### Procedural details

**FIGURE 1:**
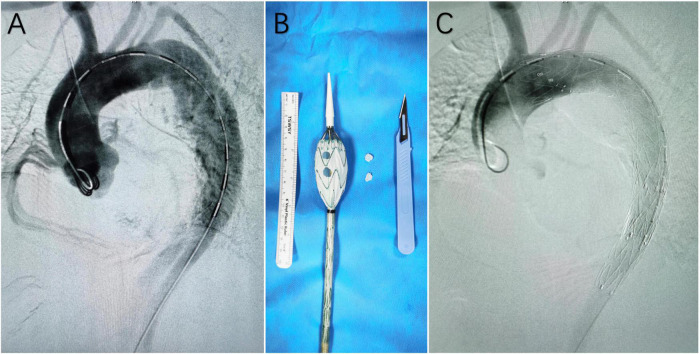
**(A)** Digital subtraction angiography (DSA) before the deployment of the fenestrated surgeon-modified stent-graft; **(B)** fenestrated surgeon-modified stent-graft; **(C)** DSA at the end of the operation, showing complete exclusion of the main entry tear and patent branch arteries of the aortic arch.

Procedural details have been described in our previous report (6). To summarize, all operations were performed under general anesthesia in a hybrid operating room. In all cases, Valiant Captivia (Medtronic, Minneapolis, MN, USA) devices were selected as the main stent graft for modification. The modification was performed on a sterile operating table. Once the stent graft was partially unsheathed, the operator would create the fenestration in the designated position with a scalpel. After the creation of fenestrations, the f-SMSGs would be resheathed with the help of assistants. Access points were the left common femoral artery for the f-SMSG, and in patients with a previous history of endovascular repair, the right common femoral artery would be used. A large sheath was introduced retrogradely through the common femoral artery. In cases where bridging stent grafts were implanted into LCCA and LSA, a sheath was introduced retrogradely through the left brachial artery into the ostium of LSA, and a sheath was introduced retrogradely through LCCA into the ostium of LCCA. After ascertaining that the fenestrations were pointing to the ground, the f-SMSG was advanced over the Lunderquist wire. On arriving at the target position, the f-SMSG was deployed under visualization. After the deployment of f-SMSG and precise alignment between fenestrations and target arteries, bridging stent grafts were advanced into fenestrations, ∼15 mm protruding into the lumen of f-SMSGs, with the remaining in the target arteries. Post-dilation would be performed for balloon-expandable bridging stent graft. When the diameter of f-SMSG at the distal landing zone was 10% larger than that of the aorta, another distal restrictive stent graft (either a bare metal stent or a covered stent, determined according to the extent of pathology) whose diameter agreed with the aorta would be deployed prior to the f-SMSG. The distal oversized part of the f-SMSG would be covered within the distal restrictive stent graft. Completion angiography would be performed to confirm that fenestrations were aligned with the target arteries and all supra-aortic trunks patent (Figure 1).

### Follow-up and definition

Follow-up surveillance was performed with serial CTA in the 6 and 12 months, and annually thereafter. No patients were lost to follow-up. Mortality, reintervention, and adverse events that occurred within 30 days after the operation or during hospitalization were reported as 30-day outcomes, otherwise were reported as follow-up outcomes. Technical success was defined as the successful alignment of all fenestrations with target arteries, patent supra-aortic trunks, and complete exclusion of primary entry tear without type I or III endoleak. Extent, chronicity classification of TBAD, and complications (entry flow, stroke) were reported according to Society for Vascular Surgery (SVS) and Society of Thoracic Surgeons (STS) Reporting Standards for TBAD (7).

### Statistics

Categorical data are reported as the absolute number and percentage; continuous data are reported as the mean ± standard deviation; and non-parametric data (e.g., follow-up time) are reported as the median and range. Statistical analysis was performed with SPSS software (22.0 v; SPSS, Inc., Chicago, IL, USA). Kaplan–Meier analysis was used for follow-up data.

## Results

From March 2016 through April 2021, 47 consecutive patients with TBAD met the inclusion criteria and underwent zone 1/2 TEVAR with f-SMSGs. There were 39 male patients (median age, 61; range: 33–77). Hypertension was the most frequently diagnosed comorbidity (*n* = 35, 76.1%), while less than half (42.6%) patients had a smoking history. Previously, the endovascular repair had been performed in two patients (4.3%). Population details are given in Table 1. All patients were diagnosed with TBAD, including 3 (6.4%) urgent operations and 44 (93.6%) elective operations. Other TBAD details are given in Table 2.

**TABLE 1:** Population demographics [median (range) or *n* (%), *N* = 47].

Age, years	61 (33–77)
Body mass index	24.46 (18.36–34.60)
Male	39 (83.0)
Hypertension	35 (76.1)
Smoking history	20 (42.6)
Coronary disease	2 (4.3)
Stroke	3 (6.4)
Diabetes mellitus	6 (12.8)
Previous endovascular repair	2 (4.3)
Chronic obstructive pulmonary disease	3 (6.4)
Renal dysfunction	4 (8.5)

**TABLE 2:** Disease details [*n* (%), *N* = 47].

Extent of pathology (B_proxiaml extent, distal extent_)	
B_1,5_	1 (2.1)
B_1,6_	2 (4.3)
B_1,9_	1 (2.1)
B_1,11_	1 (2.1)
B_2,4_	4 (8.5)
B_2,6_	2 (4.3)
B_2,8_	2 (4.3)
B_2,9_	2 (4.3)
B_2,10_	2 (4.3)
B_2,11_	4 (8.5)
B_3,4_	8 (17.0)
B_3,5_	4 (8.5)
B_3,6_	3 (6.4)
B_3,9_	7 (14.9)
B_3,10_	1 (2.1)
B_3,11_	3 (6.4)
Chronicity	
Acute (1–14 days)	26 (55.3)
Subacute (15–90 days)	12 (25.5)
Chronic (>90 days)	9 (19.1)

**FIGURE 2:**
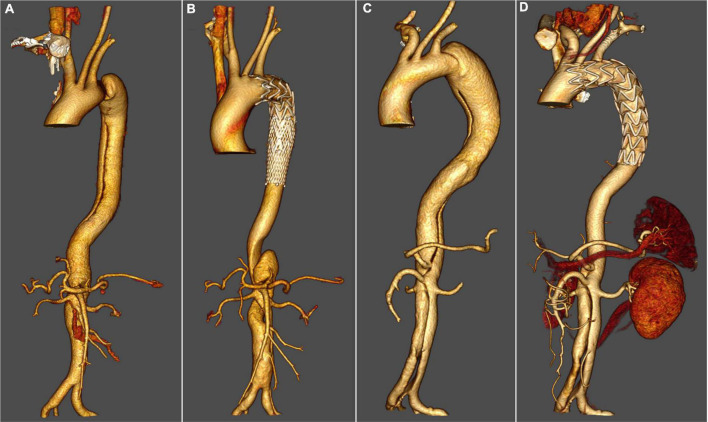
Thoracic endovascular aortic repair with fenestrated surgeon-modified stent graft. **(A)** Pre-operative three-dimensional computed tomography angiography (CTA) reconstruction for a patient with zone 1 TEVAR. **(B)** Three-dimensional CTA reconstruction for a patient with zone 1 TEVAR 3 years after the operation. **(C)** Pre-operative three-dimensional CTA reconstruction for a patient with zone 2 TEVAR. **(D)** Three-dimensional CTA reconstruction for a patient with zone 2 TEVAR 4 years after the operation.

In total, 21 zone 1 and 26 zone 2 TEVAR were performed. A total of 65 arteries were revascularized successfully with fenestrations, including 21 LCCA, 41 LSA, 1 RCCA that originated directly from the aortic arch, 1 aberrant right subclavian artery (aRSA), and 1 aberrant left vertebral artery (LVA) that originated directly from the aortic arch. LCCA–LSA bypass surgery was performed in one patient (in the same stage). Distal restrictive stent grafts were used in 25 patients, including 15 Sinus-XL (OptiMed Medizinische Instrumente GmbH, Ettlingen, Germany), 9 Hercules (MicroPort, Shanghai, China), and 1 Wallstent (Boston Scientific, Boston, MA, USA). Technical success was achieved in 46 patients (97.88%). In one patient, the f-SMSG migrated during deployment and the fenestration was misaligned with LSA, and LSA was revascularized successfully with the chimney technique. Fluency (CR Bard, Murray Hill, NJ, USA) was the mostly used bridging stent-graft (*n* = 12), and then was Viabahn (WL Gore, Flagstaff, AZ; *n* = 5) and E-luminexx (CR Bard, Murray Hill, NJ, USA; *n* = 3) (Figure 2).

### Thirty-day outcomes

The mortality rate was 2.1% (*n* = 1). The patient died of retrograde type A aortic dissection (RTAD). When a sudden drop in blood pressure was recorded, he was sent for surgery immediately. A newly occurred tear was found in the aortic arch (lesser curvature side). The ascending aorta and the aortic root were dissected, and the blood flow of the right coronary artery originated from the false lumen. The estimated survival (± SE) at 30 days was 93.6 ± 1.0% (95% Confidence Interval [CI], 92.6–94.6%). No stroke was observed. The rate of reintervention was 6.4% (*n* = 3). RTAD was the reason for reintervention. Two patients had zone 1 TEVAR and had aortic root reconstruction, ascending aorta, and total aortic arch replacement, and endovascular repair of the descending aorta. Another patient had a Bentall procedure, transposition of the aortic arch, and frozen elephant trunk implantation performed because of RTAD and severe aortic insufficiency. The estimated freedom from reintervention (± SE) at 30 days was 91.5 ± 1.2% (95% CI, 90.3–92.7%).

### Follow-up outcomes

**FIGURE 3:**
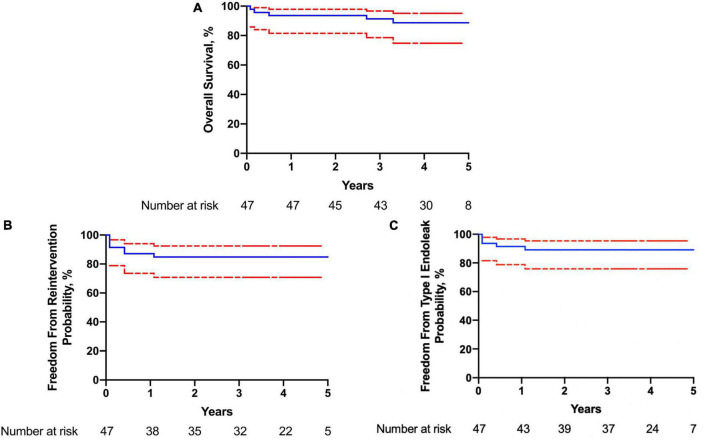
Kaplan–Meier curve of **(A)** overall survival; **(B)** freedom from reintervention; **(C)** freedom from type I endoleak.

The compliance of imaging follow-up at 6 months, 1, 2, and 3 years was 91.5% (43/47), 59.1% (26/44), 47.6% (20/42), and 33.3% (13/39), respectively. Clinical follow-up with phone or outpatient visits was performed each year for all patients. During follow-up, there were four deaths recorded. One patient had a rupture of AD 2 months after the operation (Figure 3). The other three patients had non-aortic related death, including one case of bilateral stroke (7 months), one case of acute coronary syndrome (26 months), and another case of cardiac arrest (38 months). The estimated survival (± SE) at 4 years was 88.7 ± 1.4% (95% CI, 87.3–90.1%). Endoleak was found in five patients (10.6%), and four patients had reintervention. One patient (1 small fenestration for LSA) had reintervention owing to type IA entry flow (5 months). Another f-SMSG aligned with LCCA was deployed proximal to the previous f-SMSG, and the entry flow was resolved. In another case, type IA entry flow was observed during the annual CTA examination, and the false lumen was embolized with a coil. Two patients had type IB entry flow, one had another covered stent graft deployed distal to the f-SMSG 5 months after the operation, and the other had a false lumen embolized with the coil. At the end of the follow-up, all supra-aortic trunks were patent. The estimated freedom from reintervention (± SE) at 4 years was 84.8 ± 1.5% (95% CI, 83.3–86.3%). Details about the 30-day and follow-up outcomes are listed in Table 3.

**TABLE 3:** Outcomes [median (range) or *n* (%), *N* = 47].

Median hospital stay	11 (5–28)
Median ICU stay	1 (0–12)
**Thirty-day outcomes**	
Mortality	1 (2.1)
**Retrograde type A aortic dissection**	
Reintervention	4 (11.8)
Retrograde type A aortic dissection	2 (4.3)
Severe aortic insufficiency	1 (2.1)
Follow-up, months	51 (16–71)
**Follow-up outcomes**	
Mortality	4 (8.5)
Aortic-related deaths	1 (2.1)
Non-aortic-related deaths	3 (6.4)
Reintervention	4 (11.8)
Type IA entry flow	2 (4.3)
Type IB entry flow	2 (4.3)

## Discussion

An adequate landing zone is an important factor influencing the outcome of TEVAR. Yoon et al. compared the outcomes of TEVAR with proximal landing zone ≥20 and <20 mm and found that <20 mm was related to a higher rate of adverse events, especially type IA endoleak (8). In some cases, when performing TEVAR for aortic arch pathologies, supra-aortic trunks would be covered for an adequate proximal landing zone (9). Revascularization of supra-aortic trunks with minimal cerebral hypoperfusion time is essential for a successful treatment, including LSA, reconstruction of which had gone through controversies, while currently there is an agreement for the necessity of its reconstruction (10). Different endovascular techniques had been used in the revascularization of supra-aortic trunks, including the chimney technique, custom-made branched stent-graft, *in situ* fenestration, and f-SMSG (11–14). Chimney technique was related to a higher incidence of type Ia endoleak owing to the gutter. Shu et al. invented a gutter-free chimney stent graft system for aortic arch dissection, while 23.1% presented immediate type IA endoleak and 7.7% type IA endoleak in a delayed fashion in their initial clinical experiment, which was still higher than the fenestrated or branched stent graft (15). Branched stent graft has the most stable design. In the early stage, custom-made branched stent-graft was used, allowing personalized treatment, while the process of measurement and manufacture took more than 1 month, preventing its usage in the emergent condition. Castor single-branched stent graft is the first off-the-shelf single-branched stent graft for the preservation of LSA in China, which had also been approved in Europe (16). However, double/triple branched stent graft was still under investigation due to the various anatomy patterns of the aortic arch, especially when more than one supra-aortic trunk needs reconstruction. Fenestrated stent graft, including *in situ* fenestration and f-SMSG, allows personalized treatment even in an emergent surgery when CTA was available. Shu et al. reported midterm outcomes of TEVAR with *in situ* fenestration with an adjustable puncture device (17). At the end of the follow-up, all supra-aortic trunks were patent, and no fractures, migrations, or bridging stent kinks were found. Our study showed similar results, all supra-aortic trunks were patent and all f-SMSGs were complete.

Compared with *in situ* fenestration, cerebral hypoperfusion time could be minimized in TEVAR with f-SMSG (18). Once the fenestration was aligned with the target arteries, the supra-aortic artery was revascularized successfully. In this series, no stroke had been observed during the perioperative period. To ensure accurate alignment, a detailed and precise pre-operative measurement is essential, including the diameter and clock position of supra-aortic trunks and their distance, and the fenestration should be designed and created accordingly. Three-dimensional printing could improve the accuracy of fenestrations. Rynio et al. compared 40 fenestrations created by vascular surgeons and found that fenestrations created in the three-dimensionally aortic template had better reliability and greater alignment with the target vessels than those based on measurements from CTA (19). Branzan et al. suggested that the three-dimensional printed aortic model could be utilized in urgent treatment as safe and feasible (20). However, during deployment, migration of the f-SMSG did occur occasionally (one out of 47 in this series, and the supra-aortic trunk was revascularized with the chimney technique). A pre-loaded guidewire has been recommended to assist in overcoming the migration (21). Chassin-Trubert et al. reported improvement in the success rate after applying the pre-loaded guidewire [from 94% (19/22) to 100% (28/28)], all of which were total endovascular aortic arch repair, revascularizing all supra-aortic trunks (22). Additionally, the alignment could be simplified and reassured with a pre-loaded guidewire, thus shortening the learning curve (23).

A major concern about f-SMSG is its durability after modification. There are studies reporting the application of Bolton (Bolton Medical, Sunrise, FL, USA), the deployment system of the Gore device (WL Gore & Associates, Inc., Flagstaff, AZ, USA), and Medtronic (Bolton Medical, Sunrise, FL, USA) as f-SMSGs (homemade fenestrated stent-graft, physician-modified fenestrated stent-graft), while none of these abovementioned devices had reported durability after modification, namely, durability after damage to the fabric in their instructions for use (IFU). Several benchtop experiments had been carried out to evaluate the safety and fabric durability after modification, and no malfunction or rapid deterioration was reported, while pathological changes bring about more sophisticated hemodynamic and biomechanical conditions (24, 25). Several studies reported promising outcomes after TEVAR with f-SMSGs for aortic arch pathologies, including type A/B aortic dissections, degenerative aneurysms, and penetrating aortic ulcers (26–28). Canaud et al. reported outcomes of total arch TEVAR with double fenestrated physician-Modified Stent-grafts for 100 patients with various pathologies. During a mean follow-up of 24 ± 7.2 months, all supra-aortic trunks were patent, and no stent-graft collapse or type III endoleak was reported (29). In our study, the median follow-up was 51 months, and although the follow-up period of five patients exceeded 5 years, no stent graft collapse or type III endoleak was observed, and all supra-aortic trunks were patent. Despite f-SMSGs’ off-label use as off-the-shelf thoracic stent grafts, their safety and durability seem acceptable in treating aortic arch pathologies.

Ma et al. have performed computation analysis to investigate the force distribution after TEVAR and found the maximal aortic stress at the apposition point between the stent graft and aorta (greater curvature side), which was also verified in an animal model (30). In our series, two out of three RTAD cases had new entry tear at the proximal end, which was bare metal stent, of the f-SMSG, where the maximal aortic stress was suggested. Zone 0/1/2/3 TEVAR had been performed in our center, and more RTAD cases were recorded in zone 1/3 TEVAR compared with zone 0/2 TEVAR. When the proximal landing zone of the stent-graft is in the extremely curved artery, the maximal aortic stress would increase greatly, and so would the risk of RTAD. It has been suggested, not only with our evidence, that zone 0 instead of zone 1–2 as the proximal landing zone in selected cases was related to lesser complications and better outcomes (31). However, zone 0 TEVAR was challenging and should only be considered in high-volume centers and performed by experienced surgeons/physicians.

## Limitations

Data were retrospectively analyzed despite being prospectively collected. The sample size was small and no control group was set to compare f-SMSG with other techniques to revascularize supra-aortic trunks, including parallel graft, branched stent graft, and *in situ* fenestration. No benchtop experiment has been performed before the clinical application of f-SMSG. Since f-SMSG is beyond the IFU, further and close follow-up is needed. All procedures were performed by an experienced surgeon.

## Conclusion

Thoracic endovascular aortic repair with f-SMSGs is a feasible alternative treatment option for TBAD involving the aortic arch. Results based on this study seem to be acceptable. Long-term safety and durability need to be assessed with a larger sample size and longer follow-ups.

## Data availability statement

The raw data supporting the conclusions of this article will be made available by the authors, without undue reservation.

## Ethics statement

The studies involving human participants were reviewed and approved by the Committee on Ethics of Medicine, Navy Medical University. The patients/participants provided their written informed consent to participate in this study.

## Author contributions

XL, LeZ, CS, HZ, SX, YY, LoZ, WG, and QL: substantial contributions to the conception or design of the work, or the acquisition, analysis, or interpretation of data for the work, and drafting the work or revising it critically for important intellectual content. All authors provide approval for publication of the content and agree to be accountable for all aspects of the work in ensuring that questions related to the accuracy or integrity of any part of the work are appropriately investigated and resolved.
